# Stereotactic radiotherapy for oligoprogressive ER-positive breast cancer (AVATAR)

**DOI:** 10.1186/s12885-021-08042-w

**Published:** 2021-03-23

**Authors:** Reem Alomran, Michelle White, Melissa Bruce, Mathias Bressel, Susan Roache, Lama Karroum, Gerard G. Hanna, Shankar Siva, Shom Goel, Steven David

**Affiliations:** 1 Peter MacCallum Cancer Centre, 305 Grattan St, Melbourne, Victoria 3000 Australia; 2 Department of Radiation Oncology, Comprehensive Cancer Centre, King Fahad Medical City, Riyadh, Saudi Arabia; 3 Monash Medical Centre, Melbourne, Australia; 4 The Sir Peter MacCallum Department of Oncology, The University of Melbourne, Parkville, Victoria 3010 Australia

**Keywords:** Oligoprogressive disease, Stereotactic radiotherapy, Advanced breast cancer

## Abstract

**Background:**

The enhanced knowledge of cancer biology has led to considerable advancement in systemic therapy for advanced breast cancer. Recently, studies showed that cyclin-dependent kinase (CDK) 4/6 inhibitor, when added to endocrine therapy, had improved the outcomes of patients with advanced ER-positive HER2-negative breast cancer. However, the disease often progresses following a period of treatment response. In a subset of patients, disease progression may occur at limited sites, i.e., oligoprogressive disease (OPD). In the past few years, stereotactic radiotherapy (SRT) has emerged as a safe and effective treatment for advanced cancer when delivered to limited metastatic sites. Hence, it is worth investigating the role of SRT in the setting of oligoprogressive breast cancer.

**Method:**

AVATAR is a multicentre phase II registry trial of SRT with endocrine therapy and CDK 4/6 inhibitor for the management of advanced ER-positive HER2-negative breast cancer. The study aims to enrol 32 patients with OPD limited to 5 lesions. The primary endpoint of the study is time to change systemic therapy measured from the commencement of SRT to change in systemic therapy. Secondary objectives include overall survival, progression free survival and treatment related toxicity. The exploratory objective is to describe the time to change in systemic therapy by the site (bone only vs. non-bone lesions) and number (1 vs. > 1) of OPD.

**Discussion:**

This study aims to explore the effect of SRT in maximising the benefit of systemic therapy in patients with oligoprogressive ER-positive HER2-negative breast cancer. This approach might help reduce the burden of disease and improve the life quality in these patients.

**Trial registration:**

ACTRN, ACTRN12620001212943. Date of registration 16 November 2020- Retrospectively registered.

## Background

Most patients with ER-positive HER2-negative advanced breast cancer commence on endocrine treatment combined with targeted therapy; reserving chemotherapy for patients with rapidly progressive disease or severe organ dysfunction. Endocrine treatment is therefore deemed the backbone of systemic therapy for most patients with ER-positive HER2-negative advanced breast cancer. However, a major challenge that limits the efficacy of this therapy is the occurrence of acquired resistance that leads to the progression of metastatic disease.

### Rationale for AI and CDK 4/6 inhibitors

The elucidation of endocrine resistance mechanisms has made it possible to identify new therapeutic targets that can be suppressed by agents such as cyclin-dependent kinase (CDK) 4/6 inhibitors [1]. The current preferred first line therapy for most patients with ER-positive HER2-negative advanced breast cancer is therefore endocrine therapy combined with a CDK 4/6 inhibitor. Several randomised controlled trials (MONALEESA-2, MONALEESA-7, PALOMA-2 and MONARCH-3) have shown an improvement in progression-free survival using this treatment combination [2–7]. Despite these significant advancements in breast cancer therapy, patients often develop disease progression necessitating the need for different strategies to improve the outcomes of these patients.

### Management of Oligoprogressive Disease

Oligoprogressive disease (OPD) is a new term that has emerged recently to describe a state in which a tumour progresses at few sites (usually 1–5 sites) while other sites remain stable or continue to respond to systemic therapy [8]. Several studies have described OPD in various cancer types [9–13], and Kelly et al. [14] systematically characterised the pattern of disease progression for patients with ER-positive advanced breast cancer receiving endocrine therapy. This study identified a subset of breast cancer patients who exhibit OPD with approximately 31% of patients showing progression (while on endocrine therapy) at < 4 sites. This situation frequently occurs in clinical practice with the extensive use of targeted therapy that may potentiate the evolution of drug-resistant subclones resulting in widespread resistance [8, 15].

Management of OPD can be challenging; however, different treatment approaches are emerging, including (1) switching systemic therapy; (2) maintaining the same line of systemic therapy if there is low burden disease progression; (3) using local ablative radiotherapy while maintaining the same systemic therapy [16]. Lately, there has been a growing interest in the use of ablative radiotherapy in the management of OPD. Indeed, several retrospective studies of patients with EGFR and or ALK mutation non-small cell lung cancer (NSCLC) treated with local ablative therapy and continued treatment with targeted therapy resulted in improved progression-free survival [17–20]. Similarly, a prospective study of stereotactic ablative radiotherapy in combination with erlotinib in patients with advanced NSCLC resulted in median progression-free and overall survival of 14.7 and 20.4 months, respectively [21]. Although Kelly et al. [14] reported that 61% of the patients identified with apparent OPD would have been suitable for ablative radiotherapy, to our knowledge, there is a paucity of data about ablative radiotherapy in oligoprogressive breast cancer.

Considerable research attention is being directed toward using local ablative radiotherapy in the management of OPD. A trial of stereotactic ablative body radiotherapy (SABR) is currently enrolling patients with oligoprogressive breast or lung cancer (NCT03808662). In this phase II study, 160 patients will be randomised to receive SABR to all OPD up to 5 lesions or standard of care which include chemotherapy or targeted therapy. This study aims to examine the role of SABR as a consolidative treatment for patients with limited OPD. The HALT trial (NCT03256981) is a phase II/III trial of SABR in patients with oncogenic driver mutation advanced lung cancer and OPD in three sites. Eligible patients will be randomised in a 2:1 ratio to receive SABR to all OPD and Tyrosine Kinase Inhibitor (TKI) or TKI alone. The primary outcome is progression-free survival (PFS) [22]. The STOP trial (NCT02756793) is a phase II study of patients with advanced NSCLC and OPD in up to 5 lesions. Patients in this study will receive standard of care therapy with or without SABR to all OPD. A phase II study of stereotactic radiotherapy (SRT) for OPD in up to 5 sites in patients with advanced renal cell carcinoma is ongoing (NCT02019576). Patients enrolled in this study will continue to receive targeted therapy. The primary endpoint of this study is 1-year local control and secondary endpoints are PFS and toxicity [23]. Also, the TRAP trial (NCT03644303) is a phase II single-arm study of SABR to OPD in advanced castrate-resistant prostate cancer. Patients will be eligible for the study if they have OPD in ≤2 sites and is limited to the prostate, lymph nodes, bones and lung. The primary endpoint is PFS and secondary endpoints include local control, overall survival (OS), toxicity and time to change to the next line of therapy [8].

### Stereotactic radiotherapy (SRT)

SABR and stereotactic radiosurgery (SRS) are advanced radiotherapy techniques that involve the delivery of high dose radiotherapy to the tumour while minimising irradiation of the surrounding normal structures [24–27]. SRS is typically delivered in single fraction while SABR is usually administered over several days. In this protocol, SRT refers to SABR and SRS.

SRT is an effective, low toxicity option for ablative management of oligo-metastases with local control > 90% and very rare grade 3 toxicities [28–30]. The NRG-BR001 is a phase I study of 42 patients with oligo-metastases, 13 of whom had breast primary, showed that SABR to 4 metastases or 2 nearby metastases is a safe treatment [31]. It is therefore rational to use SRT to ablate the progressing disease sites in oligoprogressive cancer in an attempt to eliminate the resistant sub-clones and to enable the continued efficacy of the systemic therapy –an AI combined with CDK 4/6 inhibitor in the present study.

The purpose of this study is to determine if SRT to five or fewer OPD sites extends the time patients with advanced ER positive HER2 negative breast cancer are treated with an AI combined with CDK 4/6 inhibitor, i.e. extends the time these patients are treated with the current line systemic therapy. Furthermore, this study will evaluate the impact of this treatment regimen on progression free and overall survival. The clinical implications of this study are particularly important given the potential for patients treated with SRT to maintain their quality of life by delaying the use of more toxic treatment for small volume progressive disease.

## Methods/design

AVATAR is a multicentre phase II registry-based study designed to assess the time to change in systemic therapy in patients with oligoprogressive breast cancer receiving an AI in combination with a CDK 4/6 inhibitor, after SRT to all oligoprogressive lesions. Secondary aims include overall survival, progression free survival and treatment-related toxicity.

Patients must have evidence of response to AI and CDK 4/6 inhibitor for a minimum of 6 months prior to study entry (defined as either stable disease or partial response).

**Fig. 1:**
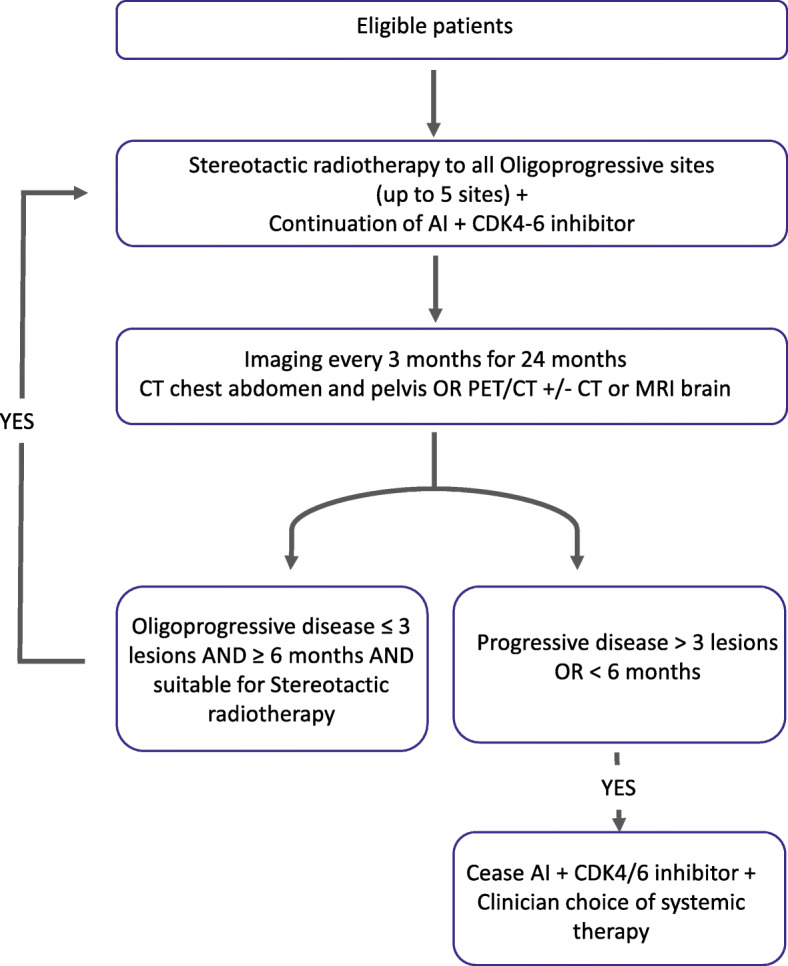
AVATAR study schema

Patients will receive SRT to all sites of OPD while continuing on AI and CDK 4/6 inhibitors. Study schema is shown in Fig. 1.

### Number of sites

The study will be activated in 8–12 sites across Australia that have the capabilities of delivering SRT. The Trans Tasman Radiation Oncology Group (TROG) will assess the suitability of each radiotherapy centre via a facility questionnaire.

### Estimated study duration

It is anticipated that 32 eligible patients will be recruited over 2 years. All participants will be followed until death or study completion. The study will be considered closed after all patients had a minimum of 2 years of follow-up or died. It is anticipated that this study will run for approximately 4 years. Participants who do not commence SRT within 28 days of enrolment will be replaced until 32 patients commence SRT.

### Primary objective

The primary objective of this study is to describe the time to change in systemic therapy after SRT in patients with ER-positive HER2-negative advanced breast cancer receiving an AI in combination with a CDK 4/6 inhibitor who have up to 5 sites of OPD.

### Secondary objective

The secondary objectives of this study are to describe:

1. Overall survival (OS)
2. Progression free survival (PFS).
3. Treatment related toxicity.

### Exploratory objective

The exploratory objective of this study is to describe the time to change in systemic therapy by the following:

1. Site of metastatic disease (bone only vs. non-bone lesions).
2. Number of oligoprogressive lesions (1 vs. > 1).

### Primary endpoint

- Time to change or cessation of systemic therapy (AI + CDK 4/6 inhibitor), defined as the time from commencement of SRT to change in systemic therapy due to any of the following events:
  - Clinically symptomatic progression requiring tumour specific palliative intervention e.g. change or cessation of anti-cancer systemic treatment, palliative radiotherapy as determined by treating doctor.
  - Development of new lesions or progression of existing lesions such that they do not meet the criteria for SRT.
  - Development of > 3 new or progressing lesions.
  - Cessation of either agent (AI + CDK 4/6 inhibitor) for more than 1 month due to any cause.
  - Development of progression in less than 6 months.

Progression will be assessed at 3-month intervals for a period of 24 months.

### Secondary endpoints

- OS assessed from date of commencement of SRT to the date of death from any cause.
- PFS assessed from date of commencement of SRT to date of first evidence of progression or death by any cause. Progression is defined as:
  - Clinically symptomatic progression requiring tumour specific palliative intervention e.g. change or cessation of anti-cancer systemic treatment, palliative radiotherapy as determined by treating doctor.
  - Development of new lesions or progression of existing lesions.
- Treatment related toxicity using the NCI Common Terminology for Adverse Events Version 5.0 (CTCAE v5.0).

### Source of participants

Potential participants will be identified from oncology clinics and Multi-Disciplinary meetings.

### Inclusion criteria

1. Male or female, > 18 years of age.
2. Patients with histologically proven ER-positive, HER2-negative advanced breast cancer receiving an AI in combination with a CDK 4/6 inhibitor. Biopsy of metastatic disease if technically feasible but not mandatory.
3. Patients must have evidence of extracranial metastatic disease with or without intracranial metastases.
4. Radiological evidence of stable or responding disease to an AI in combination with a CDK 4/6 inhibitor for a period of at least 6 months prior to study entry. (Patient must have ongoing stability/response in at least one lesion at the time of registration).
5. Evidence of new or existing OPD in 1–5 lesions. With reference to RECIST 1.1 [32] and or PERCIST 1.0 [33], OPD is defined as follow:
  - Using CT for intracranial or extracranial OPD:
    i. > 5 mm increase in the diameter of an existing lesion OR
    ii. > 20% increase in the diameter of an existing lesion on 2 consecutive imaging studies at least 2 months apart OR
    iii. The appearance of one or more new soft tissue lesions, measuring > 5 mm.
  - Using Positron Emission Tomography for extracranial OPD:
    i. > 30% increase in 18F-FDG SUV peak, with > 0.8 SUV units increase in tumour SUV from the baseline scan in pattern typical of tumour and not of infection/treatment effect OR
    ii. New 18F-FDG avid lesions typical of cancer (including new bone lesion) and unrelated to treatment effect and/or infection.
6. For patients with liver or lung metastases, maximum of 3 oligoprogressive lesions in single organ.
7. All OPD must be amenable to SRT.
  - Patients with risk of bone fracture are not candidate for SRT
8. For patients with intracranial metastases, SRT is the preferred treatment option, however, if clinical assessment indicated that upfront surgery is better, then post-operative SRT to the surgical cavity is recommended.
9. ECOG performance status 0–2.
10. Life expectancy ≥6 months.
11. Provision of written informed consent.
12. Clinician and participant are willing to continue current line of therapy.

### Exclusion criteria

1. Pregnancy or lactation at the time of study entry.
2. Evidence of more than one clone of metastatic disease e.g. ER-positive and ER-negative and or HER2-positive disease. (To exclude those with some ER-positive and some TNBC or HER2-positive disease).
3. Evidence of Leptomeningeal disease.
4. Evidence of Malignant cord compression.
5. Previous chemotherapy for metastatic disease (chemotherapy for primary breast cancer is allowed).
6. Contraindication to radiotherapy.
7. Previous radiotherapy in which the treated area planned to receive treatment is greater or equal to EQD2 40 Gy.
8. Previous SRT to an oligoprogressive lesion.
9. Any condition deeming the patient unsuitable to comply with the study.

### Participation in other clinical trials

Patients must not be enrolled on other clinical trials involving anticancer treatment, without the agreement of the Coordinating Principal Investigators (CPIs) of both studies, and if the other study has a therapeutic intervention, the patient must be beyond the primary endpoint of that trial and not continuing to receive study treatment.

### Registration

REDCap application will be used to develop the study database to collect patient information. The database will be used to collect patient information directly without using paper CRFs.

### Intervention

#### Dose prescription and fractionation

Table 1 shows different fractionation schedule based on SABR-COMET-10 protocol [34]. Biologically effective dose (BED) is calculated assuming α/β of 4. Preferred fractionation schedule is 20 Gy in single fraction when feasible. However, alternate fractionation can be considered as per the treating radiation oncologist.

**Table 1:** Dose prescription and fractionation to be used for patients on the AVATAR study

Preferred dose (Gy)	Number of fractions	BED_**4**_ (Gy)	Acceptable range Doses (Gy)	BED_**4**_ (Gy)	Frequency
20	1	120	16–24	80–168	–
30	3	105	24–33	72–123.75	Daily
35	5	96.25	25–40	56.25–120	Daily

#### Treatment timelines

Patients must commence SRT within 28 days of study registration.

Patients must complete all SRT treatments within 21 days period. SRT treatment should be delivered during CDK4/6 inhibitor week off if feasible.

The treating doctor should ensure that patients stop their CDK4/6 inhibitor at least 3 days prior to their commencement of SRT. Once the radiation is complete, patients can resume their CDK4/6 inhibitor 3 days later.

#### Target volumes

- Gross Tumour Volume (GTV): GTV include the gross tumour as determined by imaging using CT or MRI or PET/CT.
- Internal Gross Tumour Volume (iGTV): Depends on tumour location. The iGTV encompass GTV in motion and contoured using MIP.
- Clinical Target Volume (CTV): Margin to account for microscopic disease depends on tumour location.
- Planning Target Volume (PTV): Uniform expansion on CTV. PTV = CTV + 1–5 mm margin.

#### Organs at risks (OARs)

OAR dose constraints are based on the SABR-COMET trial protocol [34].

#### Quality assurance

Quality assurance of radiotherapy will be conducted by TROG via site questionnaire.

### Study assessments

#### Schedule of assessments

All study assessments must be performed once the patient has provided informed consent for study participation as shown in Table 2.

**Table 2:** AVATAR schedule of assessments

	Baseline	Every 12 weeks
Informed consent	X	
Medical History	X	
ECOG performance status	X	X
CT chest, abdomen and pelvis or FDG PET/CT confirming oligoprogressive disease	X	
Tumour Evaluation		X
Treatment-related toxicity assessment		X
Switching/cessation of systemic therapy		X
Further Stereotactic radiotherapy		X

#### Three monthly follow-up

Patients will be followed at 3 monthly intervals. At each visit, the investigator will assess the patient for the following:

- ECOG Performance status.
- Tumour Evaluation:
  - ◦ CT Chest, Abdomen and Pelvis OR FDG PET/CT (Investigator to keep imaging consistent for each patient).
  - ◦ For patients who have had a bone scan showing progressive disease without CT changes, FDG PET/CT is recommended to confirm progression.
  - ◦ High contrast CT or MRI brain (if clinically indicated).
  - ◦ MRI spine (if clinically indicated).
- Treatment related toxicity assessment as per NCI CTCAE v5.0

#### Management of Further Progression

Upon further progression, a patient can be candidate for further SRT to all oligoprogressive sites if all the following criteria are met:

- Evidence of OPD in 1–3 sites.
- Evidence of progression ≥6 months period.
- All oligoprogressive lesions are amenable for SRT. Reirradiation with SRT to a previously treated lesion is not permitted.

Patients who do not meet the abovementioned criteria will be deemed to have progressive disease and will no longer be followed as per the trial protocol.

### Safety reporting

#### Adverse events

An Adverse event (AE) is any unfavourable effect in a patient or research participant offered a medical treatment or intervention that does not necessarily have a causal association with this treatment or intervention.

#### Reporting of adverse events

AEs will be reported as part of the clinical registry and will include the name of the adverse event, the worst grade, causality and whether it is related to study treatment.

### Statistical considerations

#### Analysis population

All eligible patients registered to the study who commenced study treatment. Patients who did not commence treatment will be replaced.

#### Statistical methods

Descriptive statistics of baseline characteristics of all treated patients will be reported. Continuous variables will be described as mean, standard deviation, median, interquartile range, minimum and maximum, and qualitative variables will be described as counts and percentages. Unless stated otherwise, the calculation of proportions will not include the missing category in the denominator. No imputation for missing value is intended and all confidence intervals provided will be 95% two-sided, unless stated otherwise.

Time to change in systemic therapy, PFS and OS curves will be described using Kaplan-Meier methods. The curves will be presented with 95% confidence intervals. Estimates at key time points (e.g. 6 and 12 months) and median times will also be provided with respective 95% confidence interval. An event history plot will also be provided. A cut-off date for follow-up will be determined at the time of analysis. The cut-off date will be chosen to enable data on follow-up to that date to be collected, where possible, on all living patients. All events occurring after this date will be ignored in the analysis in order to minimise reporting bias.

Safety will be assessed using CTCAE v5.0 and the maximum toxicity grade per patient of each adverse event will be derived and presented in table format. The number of patients who suffer from grade 3 or higher toxicities (for each toxicity type and overall) will be provided.

#### Sample size

We consider SRT to not provide a clinically meaningful benefit in this setting if ≥75% of patients require a change in systemic therapy within 6 months of enrolment (i.e. < 25% of patients remain on AI + CDK 4/6 inhibitor 6 months after SRT). The percentage of patients requiring a change to therapy at 6 months is expected to be 50%. With those assumptions, 32 patients will allow the rejection of the null hypothesis of 25% if the true rate is 50% with > 80% power and 2.5% one sided alpha assuming up to 3 patients may not be evaluable (censored) for the primary endpoint.

#### Analysis plan

Only one analysis is planned for the study. The analysis will be performed at the completion of the study assessing all trial endpoints.

### Confidentiality

All information relating to study participants must be handled with strict professional confidence. Identifiers of any study participants should not be disclosed to anyone not directly involved in the study or the clinical care of that participant.

### Data protection

All data collected for study purposes will be securely stored on central servers, the WEHI, or per local institutional requirements. Access to the study data will be restricted to trial staff. In addition, Site Trial Coordinators will only have access to their own centre’s data. At the Trial Coordinating Centre, a password system will be utilized to control access to the trial database, and only those staff members working on this study will be allocated access to this trial. All reports prepared will be prepared such that no individual subject can be identified. All trial data required for the monitoring and analysis of the study must be completed and entered on the REDCap eCRF. Data corrections will be performed according to the instructions provided. It is the responsibility of investigational sites to maintain all source documents related to the trial which may include hospital records, the investigator’s participant study files, and the results of investigations such as blood tests and imaging.

The following information must be documented in the participant’s medical record:

- The participant’s protocol identification
- The participant number and the date of participation in study
- A notation that informed consent was obtained and the date of the consent
- Medical history
- Dates of all participant visits and results of essential trial parameters
- Occurrence and status of any AEs
- The date the participant exited the study. In addition, a note indicating if the participant completed the study or discontinued it and the cause of discontinuation

All study-related documentation at sites will be maintained for at least 15 years following completion of the study.

## Discussion

Nearly one third of patients with ER-positive advanced breast cancer will develop OPD while on endocrine therapy [14]. However, OPD is expected to be encountered more frequently in the clinic with the increased use of endocrine therapy combined with CDK 4/6 inhibitor that could trigger resistance and disease progression [8, 15]. Therefore, SRT may be a promising treatment modality to overcome resistant breast cancer without significant toxicity.

AVATAR is a multicentre phase II registry trial that aims to examine the SRT’s role as a treatment option for patients with oligoprogressive ER-positive HER2-negative advanced breast cancer. This study aims to accrue 32 patients and examine the impact of SRT on maximizing the benefit of systemic therapy, overall survival, progression free survival and toxicity.

Limitations of this study include the small sample size and single-arm design. Furthermore, the assessment of further progression following treatment intervention will not be based on RECEIST 1.1 or PERCIST 1.0 criteria. The results of this study will help design a phase III study in the future and improve our knowledge about oligoprogressive breast cancer.

## Data Availability

Not applicable.

## Abbreviations

AE: Adverse events
AI: Aromatase inhibitor
ALK: Anaplastic lymphoma kinase
BED: Biologically Effective Dose
CDK: Cyclin-dependent kinase
CT: Computed tomography
ECOG: Eastern Cooperative Oncology Group
EGFR: Epidermal growth factor receptor
ER: Estrogen receptor
FDG: Fluorodeoxyglucose
HER2: Human epidermal growth factor receptor 2
HREC: Human Research Ethics Committee
RGO: Research Governance Office
MRI: Magnetic resonance imaging
MIP: Maximum Intensity Projection
OPD: Oligoprogressive disease
OS: Overall survival
PERCIST: Positron Emission tomography Response Criteria In Solid Tumours
PET: Positron emission tomography
PFS: Progress free survival
RECIST: Response Evaluation Criteria in Solid Tumours
SRT: Stereotactic radiotherapy
SUV: Standardized uptake value
TNBC: Triple negative breast cancer
TKI: Tyrosine kinase inhibitor
TROG: The Trans Tasman Radiation Oncology Group

## References

[1] Hart CD, Migliaccio I, Malorni L, Guarducci C, Biganzoli L, Di Leo A (2015). Challenges in the management of advanced, ER-positive, HER2-negative breast cancer. Nat Rev Clin Oncol.

[2] Finn RS, Martin M, Rugo HS, Jones S, Im S-A, Gelmon K, Harbeck N, Lipatov ON, Walshe JM, Moulder S, Gauthier E, Lu DR, Randolph S, Diéras V, Slamon DJ (2016). Palbociclib and letrozole in advanced breast cancer. N Engl J Med.

[3] Hortobagyi GN, Stemmer SM, Burris HA, Yap YS, Sonke GS, Paluch-Shimon S, Campone M, Petrakova K, Blackwell KL, Winer EP, Janni W, Verma S, Conte P, Arteaga CL, Cameron DA, Mondal S, Su F, Miller M, Elmeliegy M, Germa C, O’Shaughnessy J (2018). Updated results from MONALEESA-2, a phase III trial of first-line ribociclib plus letrozole versus placebo plus letrozole in hormone receptor-positive, HER2-negative advanced breast cancer. Ann Oncol.

[4] Goetz MP, Toi M, Campone M, Sohn J, Paluch-Shimon S, Huober J, Park IH, Trédan O, Chen SC, Manso L, Freedman OC, Garnica Jaliffe G, Forrester T, Frenzel M, Barriga S, Smith IC, Bourayou N, di Leo A (2017). MONARCH 3: abemaciclib as initial therapy for advanced breast cancer. J Clin Oncol.

[5] Di Leo A, O’Shaughnessy J, Sledge GW, Martin M, Lin Y, Frenzel M (2018). Prognostic characteristics in hormone receptor-positive advanced breast cancer and characterization of abemaciclib efficacy. NPJ Breast Cancer.

[6] Johnston S, Martin M, Di Leo A, Im S-A, Awada A, Forrester T (2019). MONARCH 3 final PFS: a randomized study of abemaciclib as initial therapy for advanced breast cancer. NPJ Breast Cancer..

[7] Tripathy D, Im SA, Colleoni M, Franke F, Bardia A, Harbeck N, Hurvitz SA, Chow L, Sohn J, Lee KS, Campos-Gomez S, Villanueva Vazquez R, Jung KH, Babu KG, Wheatley-Price P, de Laurentiis M, Im YH, Kuemmel S, el-Saghir N, Liu MC, Carlson G, Hughes G, Diaz-Padilla I, Germa C, Hirawat S, Lu YS (2018). Ribociclib plus endocrine therapy for premenopausal women with hormone-receptor-positive, advanced breast cancer (MONALEESA-7): a randomised phase 3 trial. Lancet Oncol.

[8] Patel PH, Palma D, McDonald F, Tree AC (2019). The dandelion dilemma revisited for oligoprogression: treat the whole lawn or weed selectively?. Clin Oncol (R Coll Radiol)..

[9] Porcelli T, Sessa F, Luongo C, Salvatore D (2019). Local ablative therapy of oligoprogressive TKI-treated thyroid cancer. J Endocrinol Investig.

[10] Triggiani L, Alongi F, Buglione M, Detti B, Santoni R, Bruni A, Maranzano E, Lohr F, D’Angelillo R, Magli A, Bonetta A, Mazzola R, Pasinetti N, Francolini G, Ingrosso G, Trippa F, Fersino S, Borghetti P, Ghirardelli P, Magrini SM (2017). Efficacy of stereotactic body radiotherapy in oligorecurrent and in oligoprogressive prostate cancer: new evidence from a multicentric study. Br J Cancer.

[11] Yu HA, Sima CS, Huang J, Solomon SB, Rimner A, Paik P, Pietanza MC, Azzoli CG, Rizvi NA, Krug LM, Miller VA, Kris MG, Riely GJ (2013). Local therapy with continued EGFR tyrosine kinase inhibitor therapy as a treatment strategy in EGFR-mutant advanced lung cancers that have developed acquired resistance to EGFR tyrosine kinase inhibitors. J Thorac Oncol.

[12] Guida M, Bartolomeo N, De Risi I, Fucci L, Armenio A, Filannino R, et al. The management of oligoprogression in the landscape of new therapies for metastatic melanoma. Cancers. 2019;11(10):1559. 10.3390/cancers11101559 PMC6826412 31615127

[13] Santini D, Ratta R, Pantano F, De Lisi D, Maruzzo M, Galli L (2017). Outcome of oligoprogressing metastatic renal cell carcinoma patients treated with locoregional therapy: a multicenter retrospective analysis. Oncotarget..

[14] Kelly P, Ma Z, Baidas S, Moroose R, Shah N, Dagan R (2017). Patterns of progression in metastatic estrogen receptor positive breast cancer: an argument for local therapy. Int J Breast Cancer.

[15] Savas P, Teo ZL, Lefevre C, Flensburg C, Caramia F, Alsop K, Mansour M, Francis PA, Thorne HA, Silva MJ, Kanu N, Dietzen M, Rowan A, Kschischo M, Fox S, Bowtell DD, Dawson SJ, Speed TP, Swanton C, Loi S (2016). The subclonal architecture of metastatic breast Cancer: results from a prospective community-based rapid autopsy program “CASCADE”. PLoS Med.

[16] Cheung P (2016). Stereotactic body radiotherapy for oligoprogressive cancer. Br J Radiol.

[17] Chan OSH, Lee VHF, Mok TSK, Mo F, Chang ATY, Yeung RMW (2017). The role of radiotherapy in epidermal growth factor receptor mutation-positive patients with Oligoprogression: a matched-cohort analysis. Clin Oncol (R Coll Radiol).

[18] Weickhardt AJ, Scheier B, Burke JM, Gan G, Lu X, Bunn PA (2012). Local ablative therapy of oligoprogressive disease prolongs disease control by tyrosine kinase inhibitors in oncogene-addicted non-small-cell lung cancer. J Thorac Oncol.

[19] Qiu B, Liang Y, Li Q, Liu G, Wang F, Chen Z, Liu MZ, Zhao M, Liu H (2017). Local therapy for Oligoprogressive disease in patients with advanced stage non-small-cell lung Cancer harboring epidermal growth factor receptor mutation. Clin Lung Cancer.

[20] Xu Q, Liu H, Meng S, Jiang T, Li X, Liang S, Ren S, Zhou C (2019). First-line continual EGFR-TKI plus local ablative therapy demonstrated survival benefit in EGFR-mutant NSCLC patients with oligoprogressive disease. J Cancer.

[21] Iyengar P, Kavanagh BD, Wardak Z, Smith I, Ahn C, Gerber DE, Dowell J, Hughes R, Abdulrahman R, Camidge DR, Gaspar LE, Doebele RC, Bunn PA, Choy H, Timmerman R (2014). Phase II trial of stereotactic body radiation therapy combined with erlotinib for patients with limited but progressive metastatic non-small-cell lung cancer. J Clin Oncol.

[22] McDonald F, Hanna GG. Oligoprogressive oncogene-addicted lung tumours: does stereotactic body radiotherapy have a role? Introducing the HALT Trial. Clin Oncol. 2018. p. 1–4. 10.1016/j.clon.2017.10.013 29153859

[23] Cheung P, Patel S, North SA, Sahgal A, Chu W, Soliman H, et al. A phase II multicenter study of stereotactic radiotherapy (SRT) for oligoprogression in metastatic renal cell cancer (mRCC) patients receiving tyrosine kinase inhibitor (TKI) therapy. J Clin Oncol. 2020;38(15_suppl):5065–5. 10.1016/j.eururo.2021.07.026 34399998

[24] Leksell L. The stereotactic method and radiosurgery of the brain. Acta Chir Scand. 1951;102(4):316–9. PMID: 14914373. 14914373

[25] Leksell L (1968). Cerebral radiosurgery. I. Gammathalamotomy in two cases of intractable pain.

[26] Churilla TM, Ballman KV, Brown PD, Twohy EL, Jaeckle K, Farace E, Cerhan JH, Anderson SK, Carrero XW, Garces YI, Barker FG, Deming R, Dixon JG, Burri SH, Chung C, Ménard C, Stieber VW, Pollock BE, Galanis E, Buckner JC, Asher AL (2017). Stereotactic radiosurgery with or without whole-brain radiation therapy for limited brain metastases: a secondary analysis of the North central Cancer treatment group N0574 (Alliance) randomized controlled trial. Int J Radiat Oncol Biol Phys.

[27] Timmerman RD, Kavanagh BD, Cho LC, Papiez L, Xing L (2007). Stereotactic body radiation therapy in multiple organ sites. J Clin Oncol.

[28] Trovo M, Furlan C, Polesel J, Fiorica F, Arcangeli S, Giaj-Levra N, Alongi F, del Conte A, Militello L, Muraro E, Martorelli D, Spazzapan S, Berretta M (2018). Radical radiation therapy for oligometastatic breast cancer: results of a prospective phase II trial. Radiother Oncol.

[29] David S (2019). Stereotactic ablative body radiotherapy (SABR) for bone only oligometastatic breast cancer: a prospective clinical trial. Breast..

[30] Palma DA, Olson R, Harrow S, Gaede S, Louie AV, Haasbeek C, Mulroy L, Lock M, Rodrigues GB, Yaremko BP, Schellenberg D, Ahmad B, Griffioen G, Senthi S, Swaminath A, Kopek N, Liu M, Moore K, Currie S, Bauman GS, Warner A, Senan S (2019). Stereotactic ablative radiotherapy versus standard of care palliative treatment in patients with oligometastatic cancers (SABR-COMET): a randomised, phase 2, open-label trial. Lancet..

[31] Chmura S, Winter K, Salama JK, Robinson C, Pisansky TM, Borges V, Al-Hallaq H, Matuszak MM, Park S, Gonzales VJ, Hasan Y, Basan JG, Wong P, Yoon HA, Horton JK, Gan G, Milano MT, Sigurdson ER, Moughan J, White JR. Phase I Trial of Stereotactic Body Radiation Therapy (SBRT) to Multiple Metastatic Sites: A NRG Oncology Study. Int J Radiat Oncol *Biology*Physics. 2018;102:S68–9. 10.1016/j.ijrobp.2018.06.187.

[32] Eisenhauer EA, Therasse P, Bogaerts J, Schwartz LH, Sargent D, Ford R, Dancey J, Arbuck S, Gwyther S, Mooney M, Rubinstein L, Shankar L, Dodd L, Kaplan R, Lacombe D, Verweij J (2009). New response evaluation criteria in solid tumours: revised RECIST guideline (version 1.1). Eur J Cancer.

[33] Wahl RL, Jacene H, Kasamon Y, Lodge MA (2009). From RECIST to PERCIST: evolving considerations for PET response criteria in solid tumors. J Nucl Med.

[34] Palma DA, Olson R, Harrow S, Correa RJM, Schneiders F, Haasbeek CJA, Rodrigues GB, Lock M, Yaremko BP, Bauman GS, Ahmad B, Schellenberg D, Liu M, Gaede S, Laba J, Mulroy L, Senthi S, Louie AV, Swaminath A, Chalmers A, Warner A, Slotman BJ, de Gruijl TD, Allan A, Senan S (2019). Stereotactic ablative radiotherapy for the comprehensive treatment of 4-10 oligometastatic tumors (SABR-COMET-10): study protocol for a randomized phase III trial. BMC Cancer.

